# Isolated Oculomotor Nerve Palsy in a Young Adult: An Unexpected Diagnosis of HIV

**DOI:** 10.7759/cureus.102620

**Published:** 2026-01-30

**Authors:** Soukaina Rachidi, Imane Abourachida, Mohamed Chraa, Nissrine Louhab

**Affiliations:** 1 Neurology, Hospital University Center Mohammed VI, Cadi Ayyad University, Marrakesh, MAR

**Keywords:** cranial neuropathy, hiv neurological manifestations, isolated oculomotor nerve palsy, neuro-ophthalmology, third cranial nerve palsy

## Abstract

Neurological manifestations of human immunodeficiency virus (HIV) infection are diverse and may occasionally present in the initial manifestation of the disease. Isolated cranial nerve palsies are uncommon and can pose a diagnostic challenge, particularly in young adults without known immunodeficiency. We report the case of a 31-year-old man who presented with headache, left eyelid ptosis, and binocular diplopia. Examination revealed an isolated left oculomotor nerve palsy with pupillary involvement. Neuroimaging excluded compressive and vascular causes, and cerebrospinal fluid (CSF) analysis demonstrated an inflammatory profile. An extensive diagnostic workup was otherwise negative, and further evaluation revealed previously undiagnosed early-stage HIV infection. Antiretroviral therapy was initiated, leading to complete neurological recovery at three-month follow-up. This case highlights that isolated oculomotor nerve palsy may represent an early neurological manifestation of HIV infection and underscores the importance of considering HIV testing in unexplained cranial nerve palsies.

## Introduction

Human immunodeficiency virus (HIV) infection is associated with a wide spectrum of neurological manifestations involving both the central and peripheral nervous systems. These complications may occur at any stage of the disease and, in some cases, precede the diagnosis of HIV infection, posing a diagnostic challenge for clinicians [1,2]. Neurological involvement can result from direct viral effects, immune-mediated mechanisms, or secondary infections, and its presentation is often heterogeneous [3].

Cranial nerve palsies represent an uncommon neurological manifestation of HIV infection and may occur in isolation or in association with other neurological signs [4]. Their clinical presentation can mimic more frequent etiologies such as vascular, compressive, or inflammatory conditions, often prompting extensive diagnostic evaluation [5]. Among cranial neuropathies, involvement of the oculomotor nerve is less frequently reported and may raise concern for life-threatening causes, including aneurysmal compression [6].

Isolated oculomotor nerve palsy itself represents a relatively frequent neuro-ophthalmological condition with a broad etiological spectrum. Population-based studies have shown that microvascular ischemia is the most common cause, followed by compressive, inflammatory, infectious, and neoplastic etiologies, particularly in older patients and those with vascular risk factors [7]. Consequently, identifying less common underlying causes may be challenging, especially in younger individuals without typical risk profiles.

Recognizing HIV infection as a potential underlying etiology in patients presenting with unexplained cranial nerve palsies is essential, particularly in young adults without known immunodeficiency or advanced disease. This report describes a case of isolated oculomotor nerve palsy leading to the diagnosis of HIV infection, highlighting the importance of systematic evaluation and early consideration of HIV testing in the diagnostic workup of focal neurological deficits.

## Case presentation

A 31-year-old man presented with a two-week history of progressive headache associated with left eyelid ptosis and binocular diplopia. The diagnostic workup was conducted over a one-week period and included early neuroimaging, followed by laboratory investigations and cerebrospinal fluid (CSF) analysis. His symptoms subsequently evolved to include anorexia and low-grade fever without documented temperatures exceeding 38 °C. There was no history of trauma, diabetes mellitus, hypertension, or vascular disease. He was not taking any regular medications and had no known drug allergies, with no previously diagnosed sexually transmitted infections. His family history was unremarkable.

On neurological examination, the patient was alert and oriented. Cranial nerve examination revealed a left oculomotor nerve palsy characterized by complete ptosis, impaired adduction, elevation, and depression of the left eye, with preserved abduction. Pupillary examination showed a dilated left pupil with reduced reactivity to light. The remaining cranial nerves were intact. Motor, sensory, cerebellar, and gait examinations were normal, and no signs of meningeal irritation were observed.

Initial laboratory investigations, including standard laboratory tests and angiotensin-converting enzyme levels, were within normal limits. A broad serologic evaluation for infectious etiologies was performed and was negative, covering testing for syphilis, hepatitis B virus, hepatitis C virus, cytomegalovirus, Lyme disease, and toxoplasmosis. Autoimmune screening, including antinuclear antibodies (ANA), extractable nuclear antigens (ENA), anti-double-stranded DNA antibodies (anti-dsDNA), and antiphospholipid antibodies, was also negative. CSF analysis revealed lymphocytic pleocytosis with a white blood cell count of 356 cells/mm³, elevated protein concentration of 1.31 g/L, and normal glucose level of 0.50 g/L, indicating an inflammatory profile. CSF infectious testing, including polymerase chain reaction and serologic analyses for syphilis, cytomegalovirus, herpes simplex virus, varicella-zoster virus, Lyme disease, Epstein-Barr virus, and *Mycobacterium tuberculosis*, was negative. Laboratory findings are summarized in Table 1.

**Table 1:** Laboratory findings at admission CSF: cerebrospinal fluid; HIV: human immunodeficiency virus; CD4: cluster of differentiation 4; CRP: C-reactive protein; ALT: alanine aminotransferase; AST: aspartate aminotransferase.

Parameters	Patient values	Reference ranges
CSF white blood cells (cells/mm³)	356	0–5
CSF mononuclear cells (%)	98	60–100
CSF neutrophils (%)	2	0–6
CSF protein (g/L)	1.31	0.15–0.45
CSF glucose (g/L)	0.50	0.45–0.80
HIV-1 plasma viral load (copies/mL)	54,300	<40
CD4 T lymphocyte count (cells/mm³)	396	500–1,500
C-reactive protein (mg/L)	<1	0–5
Serum creatinine (mg/L)	9.98	6–13
Serum urea (g/L)	0.25	0.15–0.45
Serum glucose (g/L)	0.83	0.70–1.10
ALT (U/L)	17	7–40
AST (U/L)	23	10–40
Total cholesterol (g/L)	1.54	1.20–2.00
Triglycerides (g/L)	0.78	<1.50

**Figure 1:**
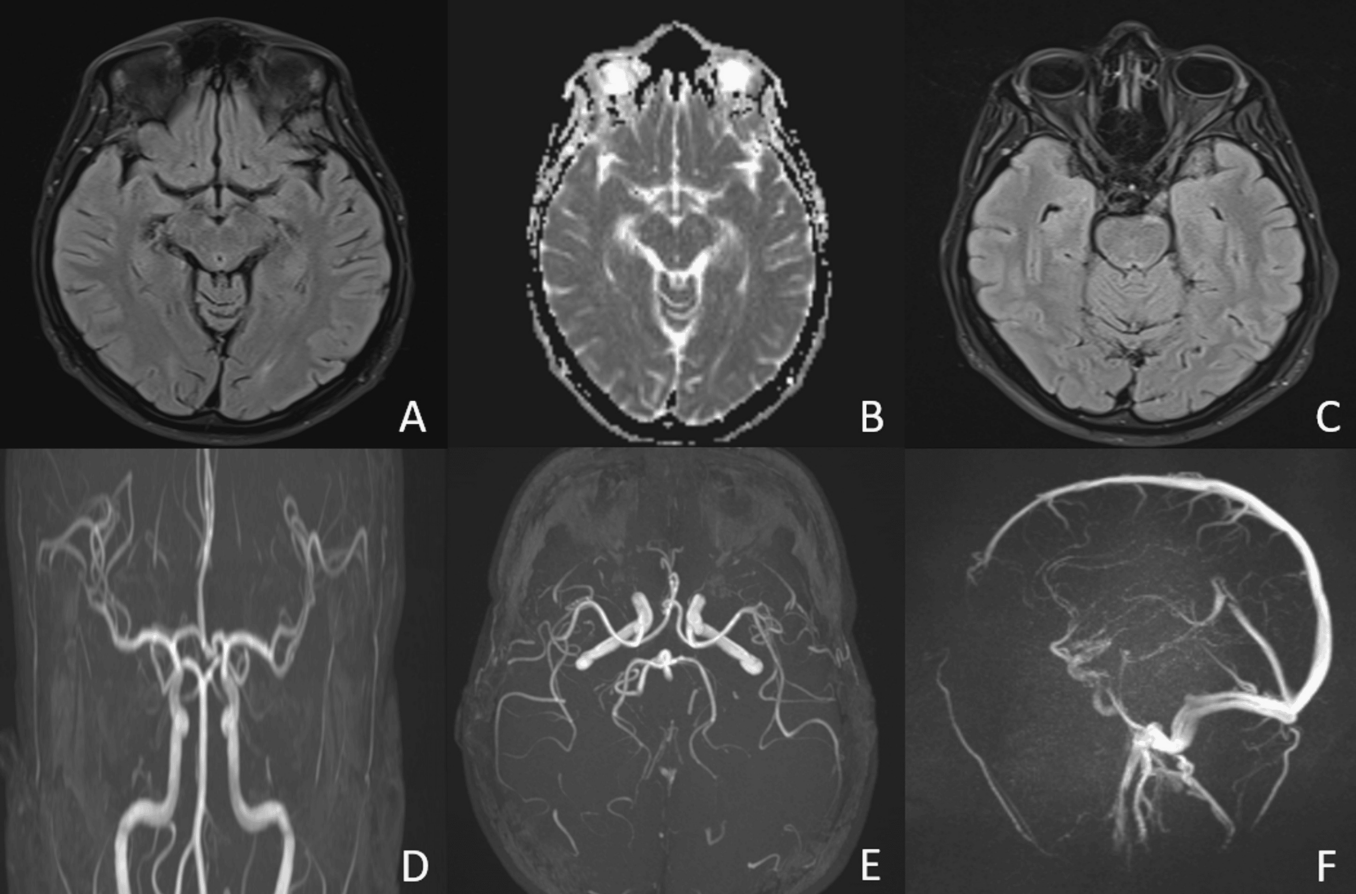
Cerebral MRI with arterial and venous angiographic sequences (A) Axial FLAIR image showing no parenchymal abnormalities; (B) Diffusion-weighted imaging showing no restricted diffusion; (C) Axial high-resolution T2-weighted image focused on the oculomotor nerve, showing no abnormality of its intracanalicular segment; (D) Time-of-flight MR angiography demonstrating normal intracranial arterial anatomy; (E) Maximum intensity projection of TOF MRA showing no aneurysm of the posterior communicating arteries; (F) Sagittal angiographic reconstruction showing no vascular abnormality FLAIR: fluid-attenuated inversion recovery; TOF: time of flight; MRA: magnetic resonance angiography

Magnetic resonance imaging of the brain showed no evidence of aneurysm, mass lesion, hydrocephalus, leptomeningeal enhancement, or parenchymal abnormalities (Figure 1).

Given the inflammatory CSF findings and the patient’s risk profile, further etiological investigation was pursued. Serologic testing for HIV was positive and confirmed by Western blot analysis, revealing a CD4 lymphocyte count of 396 cells/mm³ and an HIV viral load of 54,300 copies/mL.

Antiretroviral therapy was initiated following completion of the diagnostic workup. The patient demonstrated progressive neurological improvement over the subsequent weeks. At the three-month follow-up, neurological examination showed complete resolution of ptosis, normalization of pupillary reactivity, and full recovery of extraocular movements, with no residual cranial nerve deficits.

## Discussion

Isolated cranial nerve palsy is an infrequent neurological presentation of HIV infection and may constitute an early neurological manifestation preceding diagnosis [1-3]. Although neurological involvement in HIV is well recognized, focal cranial neuropathies occurring in isolation remain less commonly reported and are often underrecognized in early infection.

The pathophysiology of cranial nerve involvement in HIV is likely multifactorial, involving direct viral neurotropism, immune-mediated inflammatory mechanisms, and para-infectious processes [4,5]. In the present case, the inflammatory CSF profile, absence of opportunistic infections, and normal neuroimaging findings argue against compressive, ischemic, or neoplastic causes and are suggestive of a presumed HIV-associated inflammatory cranial neuropathy. Microvascular ischemic palsy was unlikely given the patient’s age and absence of vascular risk factors. Tolosa-Hunt syndrome and neurosarcoidosis were considered but were not supported by imaging, laboratory findings, or clinical course. Notably, the preserved CD4 lymphocyte count supports previous observations that focal neurological manifestations may occur early in the course of HIV infection, independent of advanced immunosuppression [3,5].

Cranial nerve palsies related to HIV infection have been described in the literature, either as isolated findings or in conjunction with other neurological manifestations [6,8]. However, many reported cases occurred in the setting of advanced disease or opportunistic infections. In contrast, our patient presented with isolated oculomotor nerve palsy as the initial neurological manifestation leading to the diagnosis of HIV infection and achieved complete recovery following initiation of only antiretroviral therapy. This favorable outcome supports the hypothesis of a potentially reversible inflammatory cranial neuropathy temporally associated with early HIV infection.

Oculomotor nerve palsy warrants particular attention because of its association with potentially life-threatening etiologies, especially intracranial aneurysmal compression. Population-based studies have demonstrated that aneurysms, neoplasms, and inflammatory conditions represent significant causes of acquired third cranial nerve palsy and must be promptly excluded, particularly in cases with pupillary involvement. In this context, comprehensive neuroimaging and targeted CSF evaluation remain essential components of the diagnostic workup [9].

## Conclusions

This case illustrates that isolated oculomotor nerve palsy can represent an initial neurological manifestation of HIV infection, even in the absence of advanced immunosuppression or known risk factors. It emphasizes the importance of a thorough and systematic diagnostic approach, including detailed neuroimaging and CSF analysis, to exclude compressive, vascular, inflammatory, and infectious causes. The favorable clinical outcome observed after initiation of antiretroviral therapy suggests that early recognition of HIV-related neurological involvement may allow for complete neurological recovery in selected cases. As this report describes a single clinical observation, these findings should be interpreted with caution and may not be generalizable. Clinicians should therefore consider HIV testing in young patients presenting with unexplained isolated cranial nerve palsies to avoid diagnostic delay and ensure appropriate management.
